# Short report: Social processing in non-emotional contexts by children with and without autism spectrum disorders (ASD)

**DOI:** 10.1371/journal.pone.0285972

**Published:** 2023-05-18

**Authors:** Elizabeth J. Teh, Melvin J. Yap

**Affiliations:** 1 Department of Otolaryngology/Division of Graduate Medical Studies, Yong Loo Lin School of Medicine, National University of Singapore, Singapore, Singapore; 2 Faculty of Arts and Social Sciences, Department of Psychology, National University of Singapore, Singapore, Singapore; Federal University of Paraiba, BRAZIL

## Abstract

**Background:**

Children with autism spectrum disorders (ASD) have been reported to show social-processing deficits in forced-choice social judgment or story interpretation tasks. However, these methods may limit examination of social-processing within a set of acceptable answers. In this pilot study, we propose a novel method predicated on the premise that language carries social information and validate this method to measure social perception in ASD.

**Method:**

20 children with ASD and 20 typically developing (TD) children matched-pairwise on age (5–12 years), gender, and non-verbal IQ, described pictures of people in everyday situations varying on extent of social engagement. Their social language production was examined in high- and low-social picture conditions.

**Results:**

The TD group produced significantly more social language in high-social than low-social picture conditions, with a large effect size (*d* = 3.15). The TD group produced significantly more social language than the ASD group under high-social conditions (*p*< .001, *η*^*2*^_p_ = 0.24), but were not significantly different under low-social conditions (*p <* .05).

**Conclusions and implications:**

The study presents proof-of-concept that expressed language carries social information. The findings indicate that social language may be used to measure social perception and examine differences in ASD, with potential applications for other clinical groups with social-processing challenges.

## Introduction

Many children with autism spectrum disorders (ASD), a neurodevelopmental condition, display characteristic difficulties with social communication. Researchers have examined social information-processing using a range of outcome measures. For example, one research paradigm requires participants to evaluate social traits such as ‘kindness’ or ‘trustworthiness’ in face-photographs using forced-choice tasks, to compare judgments made by people with and without ASD [1,2]. Another method involves presenting social scenes or story vignettes to participants and asking them to describe the scenario and social intentions of characters to code their responses on the type of social attributions and decisions made [3,4]. Using such methods, researchers have reported common deficits in social processing in individuals with ASD, compared to typically developing (TD) groups. These deficits include atypical social judgments, more errors in encoding social cues, and more avoidant responses [1–4]. However, a limitation of these measures is that there may be a constrained set of ‘acceptable’ answers, which potentially disregards other responses and/or fails to show the other socially-relevant cues that may be processed instead. Another limitation is that coding involves a degree of subjective interpretation of participants’ responses. The present study aims to pilot a new method of language analysis using empirically-derived social language ratings, which will facilitate a more objective examination of the extent to which children with ASD process socially-relevant stimuli by analyzing their discourse.

### Proposed social language analysis

There is well-established research that words carry emotional valence information [5], and this principle has been applied to study emotional processing in ASD [6,7]; for a review, see Lartseva et al., 2015 [8]). For example, Losh and Capps [6] analysed emotional discourse produced by children with ASD and found that they are less likely than TD counterparts to connect emotions to socially-based reasons. An example of a statement where an emotion is connected to a social relationship or situation is given in Losh and Capps ([6], page 812), *“I felt embarrassed because everyone laughed at me*.*”*) Teh et al. [9] counted the frequency of emotional terms produced in picture descriptions by children with ASD and TD children under systematically-manipulated social and valence conditions. They reported that higher social engagement in pictures inhibited the production of emotional terms for positive pictures only in ASD groups, while it increased the production of emotional terms for negative pictures only in TD groups. Both studies’ findings are consistent with Happé and Frith’s [10] theory that social cognition components such as agent-identification, social hierarchy mapping, and emotional processing, could potentially be separable processes in individuals with ASD and other neurodevelopmental disorders. Specifically, there may be breakdowns in inferring social roles (agents), the nature of actions or interactions, mental states, and/or contextual settings. Thus, we propose to pilot a social language measure that will capture the foregoing components for closer examination.

Whereas Warriner et al.’s study [5] rated word-valence from negative to positive, there is now evidence that words also carry a range of social content. For example, Diveica et al. [11] collected ‘socialness ratings’ for 8,948 English words by asking participants to rate the degree to which words had social relevance in terms of person characteristics, behavior or interaction, social role, space or institutions/system, social value or any other socially-relevant concept. Thus, they found that words ranged from 1 (low; e.g. *eucalyptus*, *horizontal*) to 7 (high; e.g. *friendship*, *socialise*). Applying this concept, we argue that a speaker’s word-choice can be used to infer social perception and interpretation of contextual cues. The referent label for a female figure in, “*The* ***woman*** *is helping a boy to put on his shirt*” versus “*The boy’s* ***mother*** *is helping him to put on his shirt*” gives differing connotations of the perceived relationship between the two people involved. (For example, while the word “*woman*” was not rated in Diveica et al. [11], norms were presented for other female figures e.g. “*mother*” = 6.67; “*businesswoman*” = 5.95; “*policewoman*” = 5.71, demonstrating variation in perceived socialness of different female roles.) To our knowledge, the extent of social processing has not been examined using word-ratings in the literature.

### Pilot study

This pilot introduces a novel social language measure for social processing in terms of social roles, state of mind, actions or interactions, and situations, representing a range of components in social cognition. To obtain a proof-of-concept, this approach will be tested using two hypotheses:-

H1: TD children will produce more social language in high-social than low-social picture conditions. This prediction is analogous to Teh et al.’s [9] report that more emotional language is produced in emotionally-valenced than in emotionally-neutral pictures. For the present study, only emotionally-neutral picture conditions will be analysed to focus on social processing without emotional processing as a confound.
H2: TD children will produce more social language than children with ASD in the high-social picture conditions but not in the low-social conditions, consistent with existing reports of reduced social information-processing in ASD.

## Method

### Power analyses

Teh et al. [9] reported a significant interaction effect of social engagement × group on emotional processing, *η*^*2*^_p_ = 0.386. Their finding was used as a proxy to conduct power analyses for the present study on social processing using a novel measure. Power calculations using G*Power 3.1.9.7 (https://link.springer.com/article/10.3758/BF03193146) indicated that a sample size of *N* = 18 would permit detection of an effect with a power of 0.80.

### Ethics statement

This study was approved by the National University of Singapore Institutional Review Board. Written informed consent was obtained from parents of all child participants prior to recruitment into the study. Additionally, verbal assent was obtained from all child participants before commencing the study tasks.

### Participants

**Fig 1:**
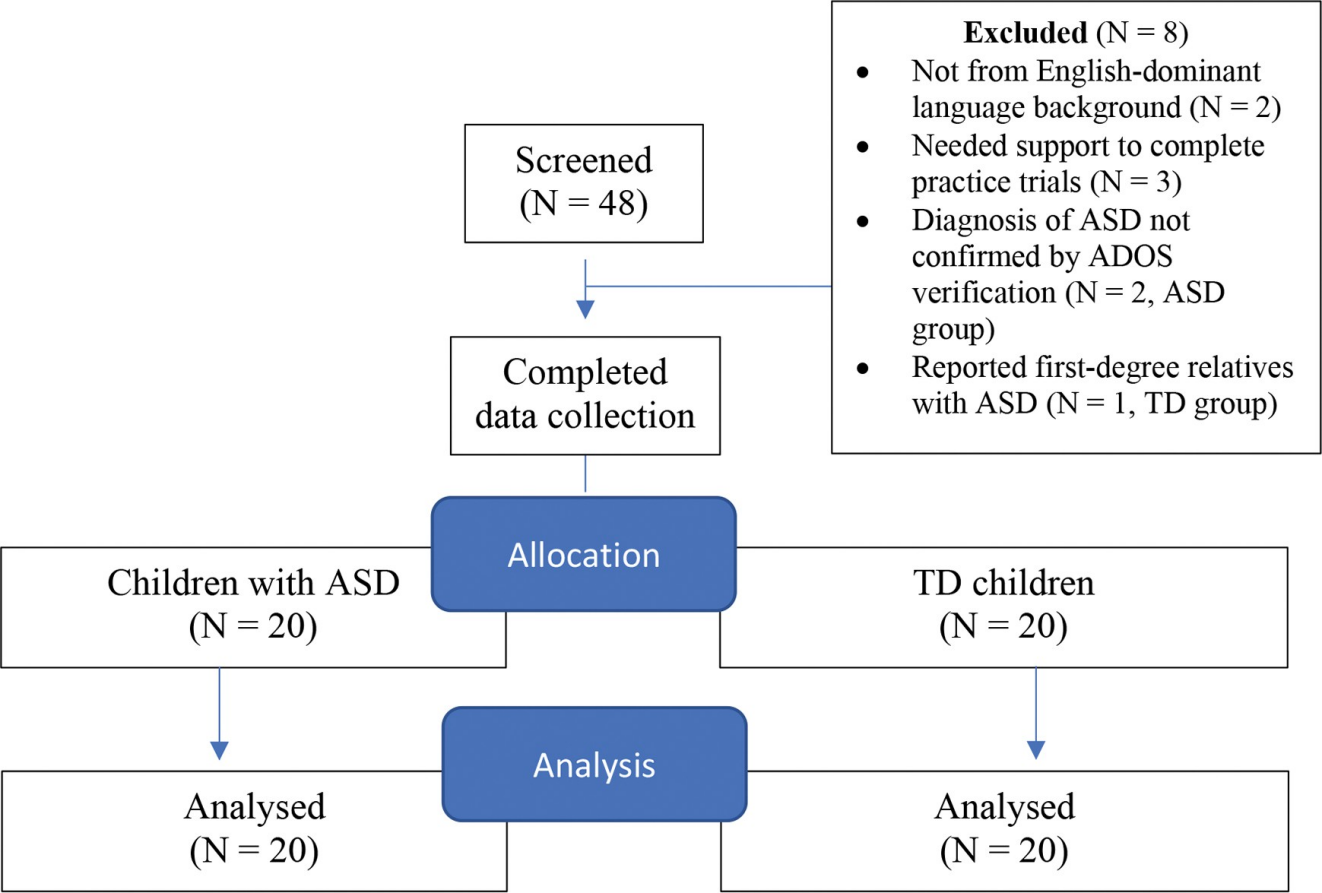
Flowchart of study recruitment.

This study uses data that was collected in Teh et al. [9] but has not been analysed for social processing, as that study examined emotional terms only. Participants were 20 children with ASD and 20 typically developing children, aged 5 to 12 years, who were individually matched on age, gender, nonverbal IQ, and socio-economic status (Table 1). For the ASD group, participants were included who had a clinical diagnosis of ASD according to parent or school reports, and were verified by experienced clinicians for this study using the *Autism Diagnostic Observation Schedule* (ADOS-2), which has reported good to excellent reliability for autism vs non-spectrum comparisons [12]. For the TD group, participants were excluded if they or a first-degree relative had a clinical diagnosis of ASD, or if the child met clinical criteria on the *Social Responsiveness Survey* (SRS-2; [13]) and/or the *Strengths and Difficulties Questionnaire* (SDQ; [14]) for conduct and emotional problems that might have an effect on social-emotional processing. Other inclusion criteria were having English-dominant language backgrounds (to minimise possible group differences in language exposure), having no known hearing or visual impairments, and being currently enrolled in mainstream education. Recruitment was conducted through schools/preschools or word-of-mouth by parents of other participants. A flowchart of the study recruitment is shown in Fig 1. Due to space constraints, details of other assessment measures can be found in Teh et al. [9].

**Table 1:** Sample characteristics.

Variable Gender	ASD (*n* = 20) 18 males, 2 females		TD (*n* = 20) 18 males, 2 females		Group comparison	
	*M*	*(SD)*	*M*	*(SD)*	*t*	*p*
Age (years)	8.10	(2.60)	8.12	(2.63)	-0.47	.65
SES	2.95	(1.05)	3.10	(0.91)	-0.59	.56
NVIQ	98.35	(13.07)	102.55	(15.16)	-1.04	.31
TOM	21.35	(6.52)	33.10	(2.85)	-6.98	< .001
CASL SS	75.35	(15.15)	108.40	(18.77)	-7.06	< .001
BLAB (English)	23.50	(5.40)	27.65	(1.96)	-3.71	.001
SRS total T-scores	69.16^a^	(11.00)	48.37^a^	(4.79)	7.70	< .001
SDQ	14.79	(5.87)	6.54	(5.03)	4.97	< .001

BLAB (English): Bilingual Language Assessment Battery (English version) raw scores; CASL SS: Standard score (*M* = 100, *SD* = 15); NVIQ: Nonverbal IQ (*M* = 100, *SD* = 15); SDQ: Strengths and Difficulties Questionnaire (higher scores indicate more difficulties); SES: Social economic status indexed by housing type (1 = 1–3 bedroom flat, to 4 = private housing) SRS: Social Responsiveness Scale (higher scores indicate more severe symptoms).

^a^ *n* = 19, one participant in each group did not return complete SRS form.

### Main experimental task

**Fig 2:**
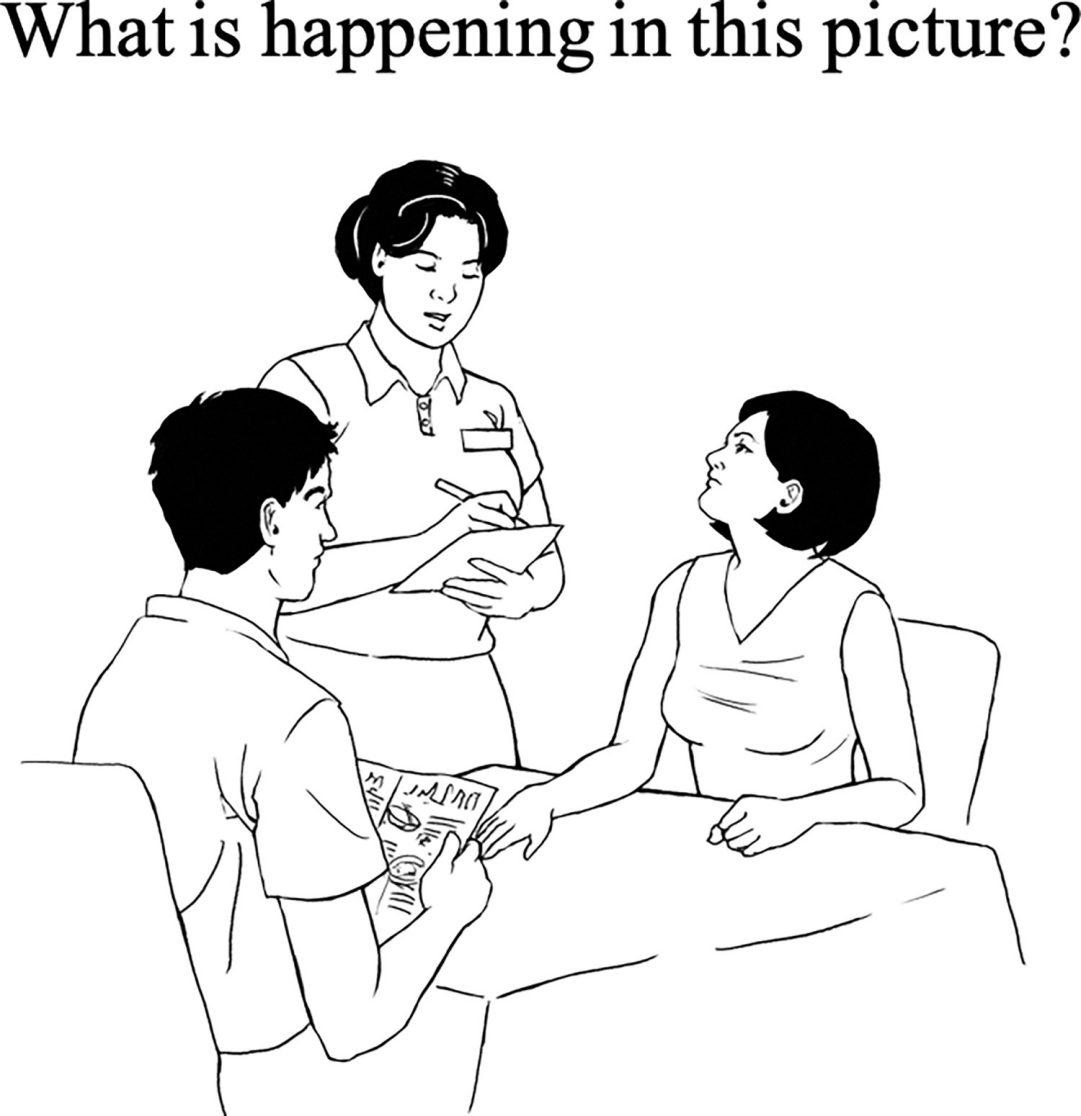
Example of an experimental trial of the picture description task.

Participants described 48 pictures presented individually on a laptop computer beneath the task prompt, *“What is happening in this picture*?*”* (Fig 2), which was also read aloud by the experimenter (first author). The present study included picture descriptions for a subset of 8 pictures representing the low-social-neutral condition (depicting one person) and 8 pictures representing the high-social-neutral condition (depicting 2 to 4 persons). More details of the experimental task can be found in Teh et al. [9].

### Coding protocol

One limitation of Diveica et al.’s [11] inventory was a lack of differentiation for words with multiple parts of speech and meanings (e.g., “*patient*” as a noun (“a sick person”) and an adjective (e.g., “tolerant”); thus, we collected new social engagement (SE) word-ratings for the present study. In an earlier unpublished study, the authors had collected picture descriptions using the standardised Pictures with Social Contexts and Emotional Scenes (PiSCES) database [15] from 20 healthy, typically developing undergraduate adults (18 females). From these descriptions, trained undergraduate research assistants then selected words with possible SE content, applying the following judgment criteria:

- a social identity or role, e.g., *doctor*, *shopkeeper*, *mother*; or
- an action which requires another person to be valid, e.g., *talking to (someone)*, *quarrelling*;
- a state-of-being or state-of-mind in relation to other people, e.g., *kind*, *helpful*, *concerned*;
- a place, situation or event where one’s behaviour may be influenced by the presence of other people, e.g., *airport*, *exam*, *wedding*.

Additionally, picture descriptions from Teh et al.’s [9] study underwent the same treatment to yield more words with potential SE content that were not found in the adults’ picture descriptions. This exercise obtained 465 words to be rated on SE content.

The ratings study was conducted online using Qualtrics (Qualtrics, Provo, UT), involving a total of 148 healthy adult participants (77 males, 71 females). Participants rated one of two non-overlapping sets of words, presented by their part-of-speech category. For nouns, participants were asked to rate the extent of social interaction between people involved in a particular place/situation (e.g., *airport*, *school*, *party*) or social role (e.g., *doctor*, *counsellor*), from *1 = ‘minimal interaction between people is implied’* to *5 = ‘significant interaction between people is implied’*. Similarly, for verbs (e.g., *talk*, *buy*) and adjectives (e.g., *patient*, *kind*), participants were asked to rate the extent to which the presence of others was implied.

Our ratings study yielded an inventory of 465 words with mean SE ratings for each word on a scale of 1–5 (*M* = 2.99, *SD* = 0.91), representing the extent to which people perceive social engagement in words. There was excellent interrater agreement on both sets of stimuli (ICC = 0.97 and 0.89), including strong agreement within each part-of-speech category (ICC = 0.66 to 0.97). Our inventory overlapped on 240 words with Diveica et al.’s [11] socialness database, and correlated strongly (*r* = .67, *p* < .001), indicating convergent validity. (Refer to S1 File for the word-ratings database).

Finally, the SE ratings were applied to the picture descriptions in the present study. For each picture description, words which carried an SE rating were assigned the respective rating, and a total SE score was calculated for each participant for each picture.

### Data analysis

Social language scores were calculated by picture conditions (high/low social engagement) and participant groups (ASD/TD). Paired *t*-tests were then applied to measure differences in social language scores of each participant group in the two picture conditions. Finally, a 2 × 2 mixed-design ANOVA was conducted, with social engagement as a within-subject factor and participant group as a between-subject factor, to compare and analyse the patterns of social language production in the different picture conditions by participant groups. All statistical analyses were conducted using SPSS version 28.

## Results

### Main analyses

The mean social engagement (SE) scores of the four participant groups in low-social and high-social experimental conditions are presented in Table 2. Firstly, the TD group obtained significantly higher social language scores in the high-social than low-social condition, *t*(19) = 14.07, *p* < .001, Cohen’s *d* = 3.15. Thus, TD children were found to produce more social language in high-social than low-social picture conditions, as predicted in Hypothesis 1.

**Table 2:** Mean social engagement scores by participant groups.

Groups	Low Social *M (SD)*	High Social *M (SD)*	Low Social–High Social comparison *(paired t-tests*, *Cohen’s d effect size)*
ASD (*n* = 20)	0.97 (1.14)	4.78 (2.46)	*t*(19) = 8.69, *p* < .001, *d* = 1.94
TD (*n* = 20)	1.00 (0.78)	7.34 (2.22)	*t*(19) = 14.07, *p* < .001, *d* = 3.15

**Fig 3:**
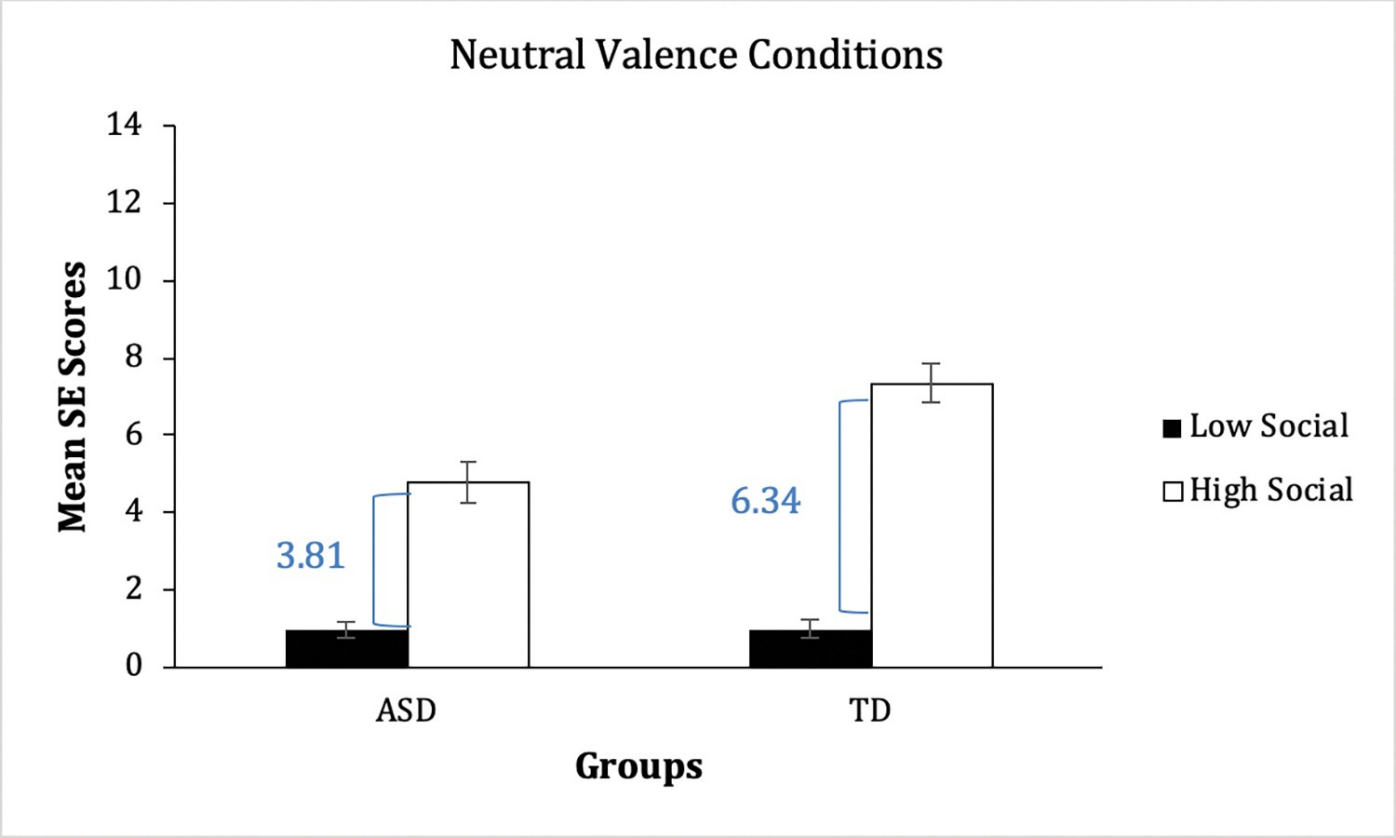
Comparison of social engagement (SE) scores within social engagement levels, in emotionally neutral conditions.

Secondly, there was a significant 2-way interaction of social engagement × group, *F*(1, 38) = 16.20, *p* < .001, *η*^*2*^_p_ = 0.30, and significant and large main effects of social engagement *F*(1, 38) = 260.47, *p*< .001, *η*^*2*^_p_ = 0.87, and group *F*(1, 38) = 7.54, *p*< .01, *η*^*2*^_p_ = 0.17. Simple effects analyses revealed a significant difference between groups in the high-social condition (*F*(1, 38) = 11.98, *p*< .001, *η*^*2*^_p_ = 0.24), but no significant group difference in the low-social condition (*p* >.05). Within the high-social condition, the increase in SE scores was numerically larger for the TD group than for the ASD group (6.34 vs 3.81; see Fig 3). Thus, TD children produced more social language than children with ASD in the high-social but not in the low-social conditions, as predicted in Hypothesis 2.

## Discussion

This study aimed to develop and validate a new method of using social language to measure social information-processing in two groups of children. The children in the TD group produced significantly more social language in high-social than low-social picture conditions, with a large effect size (*d* = 3.15), providing a proof-of-concept that expressed language carries social information. Further, under high-social conditions, the TD group produced significantly more social language than the ASD group, while the two groups produced similar (i.e., negligible) levels of social language under low-social conditions. These findings suggest a deficit in processing social information in ASD, which is consistent with findings from other studies using face-photographs or narrative paradigms to compare social processing in groups with and without ASD [1–4]. Moreover, our findings parallel Teh et al.’s [9] study which reported that emotional processing by children with ASD was lower in high-social-and-emotional than low-social-and-emotional picture conditions. The results provide further evidence supporting the use of this measure to assess social processing under different conditions. We discuss below how social language has potential applications for measuring social processing in children with ASD and other groups with challenges in social cognition.

### Social language as a measure of social processing

Researchers have applied the use of emotionally-valenced words to infer affective processes in normal and clinical populations [e.g., 5–9]. In a similar vein, we demonstrated that the social content of words can be used to infer social processes. Going further, whereas emotion research commonly used frequency or proportion of emotional words to measure processing, our pilot study applied word-ratings on a scale of 1 to 5 to better indicate the extent of social engagement processed. This approach provides a way to compare social processing in a continuous way that can account for a range of skills, addressing a limitation in other methods that focus on presence/absence or ability/impairment in social processing research. We propose that this approach is promising as a measure of social cognition to facilitate greater understanding of variable abilities across contexts, age-groups, or time-periods in future research.

### Implications for ASD research

In the present study, the smaller change in scores between low- and high-social conditions for the ASD than TD group suggests that sensitivity to social content was present but attenuated in the ASD group. Some differences between the picture conditions in this study were in the presence or absence of social interaction, types of social roles, and contexts depicted. Evidently, children with ASD tended to extract less social information than their matched TD counterparts. One potential reason is that processing of social scenes requires complex integration of multiple contextual cues and background knowledge, but individuals with ASD may have selective complex-processing impairments [16]. However, the small number of picture scenarios used in this study limited exploration of group differences in processing of specific contextual cues. Further work with a larger stimulus dataset would facilitate a more fine-grained analysis of the various social components to illuminate where the breakdown(s) lie for children with ASD.

Conversely, another advantage of this method is that language samples display areas of relative abilities that the child is processing successfully. Our social language measure will facilitate a more detailed, accurate analysis of which cues are perceived and how they are being interpreted, as well as cues that have been missed out. In turn, this provides a pathway for designing targeted interventions based on need, e.g., to identify people’s roles from specific contextual cues. Such interventions may facilitate generalization to improve daily social functioning in children with ASD.

### Future directions

We propose that social language will be useful for researchers and professionals working with clinical populations (e.g., speech therapists, psychologists) to analyse the actual language produced for both abilities and deficits in social processing. Further research is needed to build a larger database of words with SE ratings given the proof-of-concept provided in this pilot study. Finally, future researchers may consider using this novel language measure with other clinical populations and indexing social processing in developmental studies.

## Supporting information

S1 File SE ratings wordlist. (XLSX)
